# Percutaneous CT-Guided Renal Cryoablation: Technical Aspects, Safety, and Long-Term Oncological Outcomes in a Single Center

**DOI:** 10.3390/medicina57030291

**Published:** 2021-03-20

**Authors:** Stefano Cernic, Cristina Marrocchio, Riccardo Ciabattoni, Ilaria Fiorese, Fulvio Stacul, Fabiola Giudici, Michele Rizzo, Maria Assunta Cova

**Affiliations:** 1 Department of Radiology, ASUGI, Ospedale di Cattinara, 30149 Trieste, Italy; 2 Department of Radiology, University of Trieste, ASUGI, Ospedale di Cattinara, 34149 Trieste, Italy; cristinamarrocchio@gmail.com (C.M.); ciabattoni.riccardo@gmail.com (R.C.); fiorese.ilaria@gmail.com (I.F.); m.cova@fmc.units.it (M.A.C.); 3 Department of Radiology, ASUGI, Ospedale Maggiore, 30125 Trieste, Italy; stacul.fulvio@gmail.com; 4 Unit of Biostatistics, Epidemiology and Public Health, Department of Cardiac, Thoracic, Vascular Sciences and Public Health, University of Padova, 35122 Padova, Italy; fgiudici@units.it; 5 Unit of Biostatistic, Department of Medical, Surgical and Health Sciences, University of Trieste, 34127 Trieste, Italy; 6 Department of Urology, University, ASUGI, Ospedale di Cattinara, 30149 Trieste, Italy; mik.rizzo@gmail.com

**Keywords:** percutaneous, renal cryoablation, interventional radiology, renal mass, technical aspects, outcomes

## Abstract

*Background and objectives:* Cryoablation is emerging as a safe and effective therapeutic option for treating renal cell carcinoma. This study analyzed the safety and long-term oncological outcomes of cryoablation in our center. *Materials and methods:* Patients who underwent computed tomography (CT)-guided percutaneous cryoablation between February 2011 and June 2020 for one or more clinically localized renal tumors were identified. Technical success and treatment efficacy were assessed. Post-procedural complications were classified according to the Clavien-Dindo system. Recurrence–free survival was determined for biopsy-proven malignant renal tumors. *Results:* A total of 174 renal tumors, 78 of which were biopsy-proven malignant carcinomas, were treated in 138 patients (97 males and 41 females, mean age: 73 years, range: 43–89 years). Mean tumor size was 2.25 cm and 54.6% of the lesions required a complex approach. Technical success was achieved in 171 out of 174 tumors (98.3%). Primary treatment efficacy was 95.3% and increased to 98.2% when retreats were taken into account. The overall complication rate was 29.8%. No complications of Clavien-Dindo grade III or more were encountered. Median follow-up was 21.92 months (range: 0.02–99.87). Recurrence-free survival was 100% at 1 year, 95.3% (95% CI: 82.1%–98.8%) at 3 years, and 88.6% (95% CI: 71.8%–95.7%) at 5 years. *Conclusions:* Cryoablation is a safe and effective technique for the treatment of small renal lesions, with no major complications when performed by expert interventional radiologists. The multidisciplinary discussion is essential, especially considering the high number of histologically undetermined lesions. Our long-term oncological outcomes are encouraging and in line with the literature.

## 1. Introduction

Renal cell carcinoma (RCC) is the most frequent renal tumor accounting for 80% of cases and it comprises 3.8% of all new cancers in Western countries, representing the 7th most common cancer in men and the 10th most common one in women [1,2,3]. In the first half of the 1980s, incidental detection of RCC showed a slight though significant increase of about 22%. Furthermore, this incidence rate disproportionally rose to 72% between 2006 and 2007 [4]. The incidence rate of RCC is still growing, with an increase of about 2.4% per year, on account of a rising number of abdominal imaging exams performed for non-renal symptoms, determining an incidental diagnosis in more than 50%–60% of cases of renal cancer [1,3,5,6,7,8,9,10]. In particular, recent years showed a considerable increase in incidental detection of the small lesions intended as masses less than 4 cm in size, typically asymptomatic and generally affecting elderly [4]. Given this large proportion of incidentally detected small lesions, considerable attention has been posed in the treatment options for these tumors. In addition, T1 cancers (<7 cm) account for more than 60% of renal tumors, T1a (<4 cm) represent 35% of cases, and T1b represent 27% of them [4], further justifying the need to explore the best and most efficacious therapy for small lesions.

Surgical intervention is still the first treatment of choice with partial nephrectomy being the gold standard treatment for T1 tumors [1,11]. Nephrectomy was historically performed with a radical intent and was then converted to a partial approach, since literature has widely demonstrated superimposable results between the two techniques with comparable five-year survival rates [7,8,12,13]. The use of partial nephrectomy is driven by the emerging need of a nephron sparing surgery, whose aims are the preservation of as much renal function as possible and the reduction of pain, morbidity, and length of hospitalization [14,15]. Given these assumptions, in the last two decades, other minimally invasive approaches emerged with thermal ablations representing the most effective and safe alternatives to partial nephrectomy for tumors <3 cm in size [1,7,8,10,16,17]. In particular, cryoablation shows meaningful advantages compared to other ablative techniques like radiofrequency ablation, such as the direct visualization of the ice ball and the large volume of treatment [2,7,14,18,19]. Despite the considerable advantages of this procedure, some noteworthy technical difficulties might hamper the technical success, mainly due to the position of the lesion or its proximity to other structures that require the change of patient’s position or the execution of maneuvers like hydro—or pneumo-displacement [12,20,21]. In addition, lesions abutting the ureter or the adrenal glands need a previous stent positioning or α-blockers administration, respectively, while upper pole lesions may require the induction of an intentional pneumothorax to avoid multiple passages across the pleura [12,19,21,22]. Moreover, the non-favorable position of the lesion might compromise the ideal insertion of the probes that should be positioned as perpendicular as possible inside the lesion itself. Despite these technical difficulties and the scarcity of long-term results in literature, oncological outcomes of cryoablation are comparable to those of partial nephrectomy [10].

In this article, we report our experience with percutaneous cryoablation of renal tumors. The first purpose of this study was to assess the technical outcomes of the procedure for all treated patients. A secondary aim was to report oncological outcomes for patients with biopsy-proven malignancy.

## 2. Materials and Methods

### 2.1. Study Design and Population

Ethical approval was obtained for this retrospective single-center study by the Local Institutional Review Board. Patients who underwent percutaneous cryoablation (PCA) under Computerized Tomography (CT) guidance between February 2011 and June 2020 for one or more clinically localized renal tumors were identified through our clinical registry and included, regardless of the histology of the lesion. Peri-renal masses treated with PCA were excluded.

Patients with a newly discovered renal mass with suspicious characteristics on imaging were evaluated by a multidisciplinary team. If, according to the current guidelines [23], PCA was considered the optimal management for the patient, interventional radiologists evaluated the feasibility of the procedure and planned the treatment. Before cryoablation, patients underwent a contrast-enhanced Ultrasound (CEUS) and a contrast-enhanced Magnetic Resonance Imaging (CE-MRI) or, if this was contraindicated, a contrast-enhanced CT (CE-CT). Relevant demographic data were collected, including age, sex, and number of kidneys. For patients’ comorbidities, the Charles Comorbidity Index was used. Lesion dimension was measured as the maximum lesion’s diameter in any plane. The location was described as anterior or posterior based on the position of the tumor relative to the kidney midline plane, which was defined by drawing a line paralleling the direction of hilar structures that bisect renal parenchyma. When a meaningful anterior or posterior designation was not possible, the descriptor “X” was assigned to the tumor, as described by Kutikov et al. [24]. The location was further specified as medial or lateral if the mass was located on the medial or lateral rim respectively. Lesions were considered near vulnerable structures if the tumor was within 1 cm from the bowel wall or within 5 mm from the ureter or renal pelvis, diaphragm, abdominal wall or psoas muscle, spleen, adrenal glands, or main vascular structures, i.e., inferior vena cava and renal artery and vein. The interposition of the costophrenic sinus along the planned cryoprobe path and the presence of cysts adjacent to the lesion were additional factors considered in this parameter. Treated tumors were classified as low, moderate, or high complexity according to the RENAL nephrometry scoring system [24].

### 2.2. Treatment Preparation

Written informed consent was obtained from all subjects after discussing the indications, benefits, and possible adverse events of the procedure.

Patients were preferably treated in the prone position. In case of anterior lesions, the oblique or lateral decubitus positions were used. The interposition of the costophrenic sinus for the treatment of tumors located at the upper renal poles was not always predictable beforehand because the interposition may not be present in the preoperative imaging. When this occurred, it required an oblique position on the same side of the costophrenic sinus or, less often, a lateral decubitus to limit lung expansion and free the path for the cryoprobe. The position was changed in case of tumor proximity to the bowel to allow its displacement. Particular attention was paid to make the patient feel comfortable to allow collaboration for the duration of the procedure. Treatment in the supine position was carried out if no other option was possible.

Once positioned, local anesthesia was administered at the planned entrance site of the cryoprobes. If needed, a dedicated anesthesiologist gave a mild sedation to the patient. General anesthesia was reserved to cases in which the patient was unable to hold the position for the duration of the procedure or could not collaborate.

Before the cryoablation, unless obtained beforehand, a simultaneous renal mass biopsy was incorporated as routine practice whenever possible, using an 18G needle and the coaxial technique. In case of very complex lesions, it was preferred to give priority to the ablative procedure to avoid immediate complications that could compromise the PCA. In patients in which multiple masses with similar imaging characteristics were treated, only a single mass was biopsied and only this one was considered a biopsy-proven malignant tumor.

### 2.3. Ablation Procedure

The procedures were performed by two interventional radiologists with 13 and more than 30 years of experience in the field, respectively. All PCA were executed under CT guidance (Toshiba Aquillion 64) and a dedicated cryostat (VISUAL ICE Cryoablation System, Galil Medical Inc., Boston Scientific) able to manage up to 10 cryoprobes simultaneously with the possibility of modifying the power for each pair of probes. A pre-ablation single portal venous phase CE-CT scan was performed to better delineate the lesion margin and any structure at risk. Unenhanced CT scans were obtained if there were contraindications to contrast medium administration.

A variable number of 17G cryoprobes were placed with 1–2 cm spacing (usually one probe for each centimeter of the lesion) in a configuration to achieve a final ice ball covering the entire tumor with a safe margin. Single or multiple scans were executed during probe positioning. The choice of the number of cryoprobes depended on the size and morphology of the lesion to be treated and on the type of cryoprobes. In our treatments, two types of probes were used, known as the 17G IceSphere and the 17G IceRod, where the former is capable of generating a more spherical ice ball and the latter is capable of generating a more elongated ice ball. The planning was based on the performance of the single probes in relation to the obtainable isotherms, considering the isotherm at −20° as effective, and on the synergistic effects linked to the presence of several needles [12,25], which depend on their spatial arrangement. In the presence of several probes, the overall ice ball, which can be assessed on CT, had to include the entire lesion and a safety margin of at least 5 mm [26]. The inclination of the cryoprobes depended on the ideal path of the needle, based on the location of the lesion, the structures present along the needle path (muscles, ribs, and genitofemoral nerve), and the abdominal structures that surround it. The need for a cranio-caudal inclination required a volumetric CT acquisition and oblique reconstructions to verify that the insertion of the needle corresponded to the expected path. Therefore, a probe with a significant cranio-caudal inclination or the use of two or more probes with a significantly different inclination, defined as an angle superior to 10°, were considered to be complex needle approaches.

In cases of lesions close to vulnerable structures in which the simple variation of the decubitus was ineffective, the hydro-dissection technique was always chosen. This technique requires the insertion under CT guidance of a 16G needle of adequate length between the lesion to be treated and the adjacent structure, more often an intestinal loop, and the injection of a variable quantity (between 50 and 300 cc) of sterile water. The needle cannula was left in place throughout the procedure for the possible redistribution of the fluids over time. In this way, it was always possible, if necessary, to perform further injections of liquid, eventually increasing the frequency of control CT scans. In some cases, in which sensitive organs were present along the course of the probe and other techniques were not an option, other maneuvers were used for needle positioning, such as a total or partial emptying of cystic lesions by inserting a 16G aspirating needle into the cyst under CT guidance. In other cases, it was necessary to pass with the probe through the liver parenchyma.

Conventional cycles consisted of a double 10-min freezing cycle, separated by a passive 9-min and an active 1-min thaw-session (double freeze thaw protocol) [9] through the use of Argon gas for the cooling phase and helium gas for the heating phase. With new generation cryoprobes, an Argon-only (single gas) system was used due to the presence of an electronic management of the heating.

Unenhanced CT imaging at 5 and 10 min was performed during positioning to monitor ice ball growth and vulnerable structures. If the ice ball did not cover the tumor, additional probes were placed. A non-contrast CT was performed after removing the probes to identify any complication. The total procedure time, measured from the initial scout image to the last control CT scan, was registered. X-ray exposure during the procedure was reported as a dose length product (DLP), measured in mGy*cm.

### 2.4. Outcomes and Follow-Up

Technical success was defined as an adequate formation of the ice ball to include the entire lesion with a safety margin of at least 5 mm. Post-procedural inpatient complications were classified according to the Clavien-Dindo system. The length of hospitalization was calculated as the number of nights spent in the Urology Department after the treatment.

All patients underwent a first CEUS within 24 h. If the treated area showed residual vascularization, a CEUS was repeated at 1, 2, and 3 weeks up to 1 month and, if the enhancement was still persistent, the post-operative CE-MRI was anticipated in order to perform a new percutaneous cryoablation. In case of a completely avascular ablated area, an MRI was performed at 6 and 12 months and annually thereafter, up to 5 years [27]. If the patient performed any contrast-enhanced imaging study that included the kidneys after five years, this was considered as the last follow-up.

Treatment efficacy was defined as a lack of enhancement of the treated lesion at one-month follow-up. Any tumor that showed residual vital tissue within this time point was considered to be a persistence. Primary treatment efficacy was calculated based on the number of persistent lesions after cryoablation. Secondary treatment efficacy was calculated after retreatment of these lesions. A recurrence was defined as an enhancing or enlarging soft tissue nodule in or around the ablation zone on cross-sectional imaging following a previously documented successful ablation.

### 2.5. Statistical Analysis

Categorical variables were reported as percentages. Continuous variables were summarized as mean ± standard deviation or median and range as appropriate, according to the data distribution. Normality of the continuous variables was tested using the Shapiro–Wilk test. Demographic information, characteristics of treated lesions, and characteristics of cryoablation treatment were compared between biopsy-proven renal carcinoma and no biopsy-proven renal carcinoma by the independent Chi-square test for categorical variables (or Exact Fischer test when appropriate), and by the student t-test or non-parametric Mann-Whitney test for continuous parameters.

Oncologic outcomes were determined only for lesions proven to be malignant renal tumors by biopsy. Recurrence–free survival (RFS) was calculated on a per-lesion basis. Overall survival (OS) was calculated on a per-patient basis. The last follow-up update was performed on 28 October 2020. Kaplan–Meier curves were used to visualize the survival distributions. Cancer-specific survival and metastasis free survival were not determined due to the limited number of events.

Statistical significance was evaluated based on a two-sided significance level of 0.05. Data analyses were conducted using R software (version 4.0.3, 2020).

## 3. Results

A total of 174 renal tumors were treated in 138 patients during the study period. There were 97 (70.3%) males and 41 (29.7%) females, with a mean age of 73 years (standard deviation [SD]: 10; range: 43–89). The Charlson Comorbidity Index was available for 131 out of 138 patients and had a mean value of 3.6 (SD: 1.5, range: 2–9). Eleven patients (8%) had a single kidney at the time of the procedure because most had a previous nephrectomy except for one patient who had congenital renal agenesis of the left kidney. An additional patient had contracted kidneys and developed a tumor on the transplanted kidney. Mean tumor size was 2.25 cm, ranging from a minimum of 0.5 cm to a maximum of 5.5 cm. Nine masses were larger than 4 cm. With regard to the tumor position within the kidney, 97 lesions were posterior (55.7%), 53 were anterior, and 24 were either at the renal poles or at the coronal plane of the kidney. Most of them were lateral (*n* = 111, 63.8%). About half of the tumors were adjacent to sensitive structures, in particular 13 were close to the renal pelvis or ureter, 15 were close to the diaphragm or covered by the costophrenic sinus, 23 were near the bowel, 17 were close to major vessels, 31 were near the abdominal wall or psoas muscles, and 20 were surrounded by cysts. Of note, one patient had a deep-seeded tumor within a polycystic kidney and a markedly reduced renal function requiring dialysis. Twelve lesions (6.9%) were deemed to be of high complexity based on their RENAL score. Seventy-eight (44.8%) of the treated lesions were biopsy-proven malignant carcinomas and 33 (19%) were benign tumors. Sixty-three (36.2%) were indeterminate lesions, either because the biopsy was not performed (*n* = 40) or because the histological analysis was inconclusive (*n* = 23). Sixteen (9.2%) of the treated lesions were recurrences after a previous cryotherapy. There were no differences in the baseline characteristics of patients and treated lesions between patients with biopsy-proven malignant carcinomas and patients with indeterminate lesions, with the exception of tumor size, which tended to be larger for malignancies.

The characteristics of the patients and renal lesions are summarized in Table 1 and Table 2.

**Figure 1:**
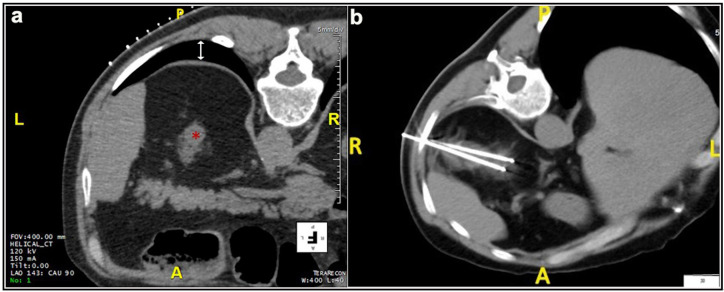
Male, 73 years old. (**a**) Preparation of the treatment of a lesion located at the upper pole of the left kidney (*). The initial scan shows the interposition of the costophrenic sinus (white arrow). (**b**) The patient was positioned in an oblique decubitus, on the same side of the costophrenic sinus, to free the path for the two cryoprobes and the lesion was adequately treated without pneumothorax.

**Figure 2:**
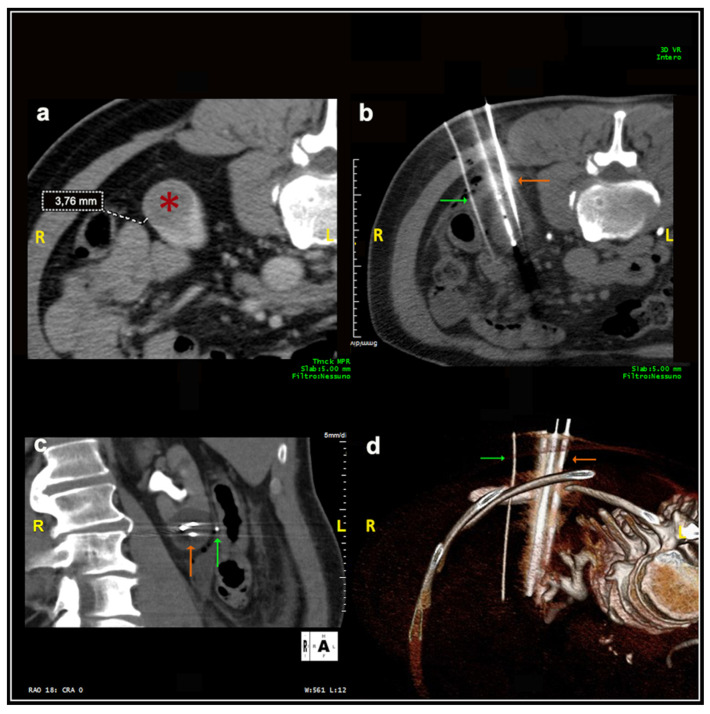
Male, 75 years old. Hydrodissection technique. (**a**) At the initial scan, the distance between the lesion (*) and an intestinal loop was 3.76 mm, significantly inferior to the safety margin of 1 cm required. (**b**) A 16G needle cannula (green arrow) was inserted for the injection of sterile water to increase the distance between the structures. The lesion was adequately treated with three cryoprobes (orange arrow). (**c**,**d**): Coronal plane and 3D reconstruction images, respectively.

**Figure 3:**
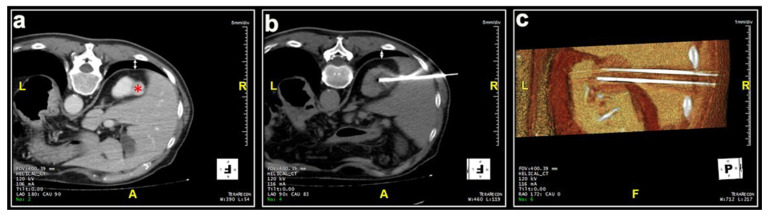
Male, 78 years old. (**a**) The initial scan shows a lesion (*) located at the upper pole of the right kidney covered by the costophrenic sinus posteriorly (white arrow) and the hepatic parenchyma laterally. (**b**,**c**) The lesion was adequately treated with two cryoprobes inserted through the hepatic parenchyma in order to avoid pneumothorax. No complications occurred.

Most patients (87.4%) were treated prone. Twenty patients were treated in the oblique or lateral decubitus position (Figure 1). Only two treatments were carried out with the patient supine. Local anesthesia was mostly sufficient for the large majority of the procedures and mild conscious sedation was administered if needed. One patient required general anesthesia for the impossibility to collaborate with breath holds during the procedure because of dementia. In most cases, one lesion was treated in each procedure. In five patients, two lesions were treated in the same procedure, and, in another two patients, three lesions and four lesions were treated in a unique procedure, respectively. A median of two cryoprobes were used for each intervention (range: 1–8). Eight cryoprobes were required for the treatment of one of the largest tumors (5.4 cm), which revealed to be a clear-cell carcinoma at biopsy. The procedure was technically successful, and no complications occurred. Eighty-nine lesions required a complex cryoprobe approach. Hydro-dissection was necessary for the treatment of 13 tumors due to the vicinity of the bowel that could not be displaced after changing the position of the patient (Figure 2). In two cases, emptying of a cyst adjacent to the lesion was deemed necessary. Only one patient required a trans-hepatic approach (Figure 3). The procedures had a median duration time of 84 min (range: 40–153). Data on radiation exposure was available for 113 out of 138 patients with a median radiation exposure of 43.7 mSv.

In Table 3, the characteristics and outcomes of the procedures are reported. There were no significant differences between patients with biopsy-proven malignant carcinomas and patients with indeterminate lesions.

The treatment was technically successful in 98.3% of cases. In one patient, the tumor was very close to the gallbladder and colon and, therefore, the freezing cycles were performed at a reduced power, which resulted in a residual vascularization of part of the lesion at the end of the treatment. One procedure was stopped due to malfunctioning of the cryoprobe during the freezing cycles. One patient was treated under local anesthesia only and did not collaborate during the procedure, especially with breath holds, which led to an incomplete treatment.

All patients were admitted for overnight observation except for one patient who was discharged a few hours after the procedure. Data on length of hospitalization was not retrievable from medical records for 13 out of 138 patients. The median length of the hospital stay was 1 day (range: 0–7). A hospitalization of 5 days or longer was required in two patients due to Clavien-Dindo II complications that required medical treatment. A longer observation period of 5 days was also necessary for a patient who had no complications but had dementia that required general anesthesia during the intervention. A total of 52 patients (29.8%) developed complications after the procedure. The vast majority of these were self-limiting perirenal hematomas of modest entity, which did not give any symptom to patients. Two cases developed a small pneumothorax that resolved spontaneously. More significant complications (Clavien-Dindo II) occurred in six patients (2.9%). None of the patients had Clavien-Dindo complications grade III or higher.

Treatment efficacy could not be assessed for two subjects, for a total of four lesions, because they did not undergo any contrast-enhanced imaging after the procedure and were immediately lost to follow-up. In addition to the three treatment failures, five more tumors had persistent enhancement at the end of the procedure (clear cell carcinomas, *n* = 2, papillary renal cell carcinoma, *n* = 1, oncocytic tumor, *n* = 1, biopsy not performed because of the complexity of the lesion, *n* = 1), with a resulting primary treatment efficacy achieved in 162 lesions out of 170 lesions (95.3%). Five persistences were successfully retreated, increasing the secondary treatment efficacy to 98.2%. Three lesions were not retreated because of refusal of the patients to undergo a second procedure.

The median follow-up for the 170 tumors was 21.92 months (range: 0.02–99.87). Thirteen lesions (7.6%) recurred including five of them within the first year, a further five within three years, and three more between three and five years of follow-up. Among recurrent lesions, five were biopsy-proven malignant tumors (clear cell carcinomas, *n* = 4, papillary renal cell carcinoma, *n* = 1). In the remaining cases, the biopsy was not performed (*n* = 6) or was inconclusive (*n* = 2). Two recurrences were tumor seeding identified within one year after the cryoablation within the posterior muscular wall, along the path of the cryoprobe. Of the 13 lesions that recurred, all underwent a second treatment except for the two patients with seeding. The second cryotherapy was successful in all cases but one, who was treated a third time and then was lost to follow-up.

**Figure 4:**
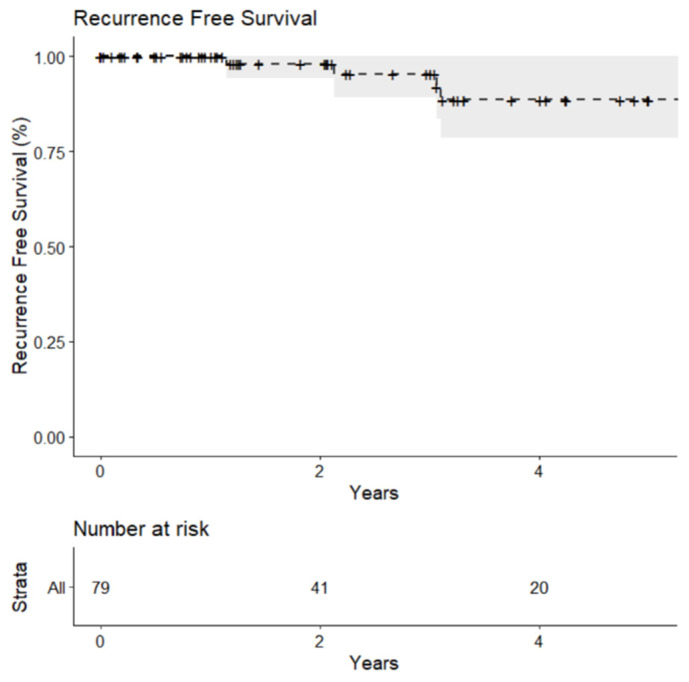
Kaplan Meier curve of recurrence-free survival for biopsy-proven malignant lesions treated with percutaneous cryoablation.

For the 78 biopsy-proven malignant lesions, the estimated recurrence-free survival at one year was 100% as none of the certainly malignant lesions had a recurrence within the first year. Recurrence-free survival decreased to 95.3% (95% Confidence Interval [CI]: 82.1%–98.8%) at three years and 88.6% (95% CI: 71.8%–95.7%) at five years (Figure 4).

**Figure 5:**
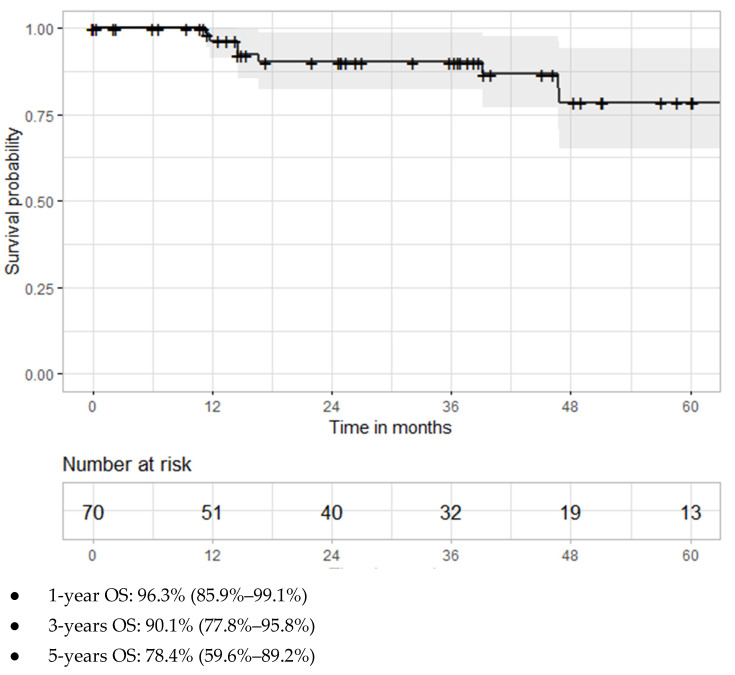
Kaplan Meier curve of overall survival for patients with biopsy-proven malignant lesions treated with percutaneous cryoablation.

Patient life status data was available for 137 of 138 patients. Median follow-up for these patients was 26.3 months (range: 0.02–96.2 months). Twenty patients died during follow-up, eight of whom were patients with biopsy-proven malignant tumors. Of the 70 patients with biopsy-proven malignant tumors, the overall survival was 96.3% (85.9%–9.1%) at one year and decreased to 90.1% (77.8%–95.8%) at three years and to 78.4% (59.6%–89.2%) at five years (Figure 5). Only two patients (one of which had a biopsy-proven malignant tumor) developed distant metastases one year after cryoablation, and, subsequently, died for the underlying renal disease about one year and one year and five months after cryotherapy, respectively. One patient had lung metastases at diagnosis and died because of renal cancer 3 years and 10 months after treatment.

## 4. Discussion

In this study, we report the technical details and long-term oncological outcomes of percutaneous cryoablation of 174 renal tumors treated in 138 patients, including 78 of which were biopsy-proven malignant carcinomas.

The literature widely discussed the technical difficulties arising from the nearness of the lesion to be treated to other structures or the necessity to use needles with a complex inclination to reach the tumor. In particular, a distance less than 1 cm between the tumor and the bowel requires a positional change of the patient to move away the bowel loop and, if this is not sufficient, pneumo-displacement or hydro-displacement are performed with carbon dioxide insufflation or saline solution injection, respectively [12,20,21]. Upper pole lesions might demand an intentional pneumothorax to avoid multiple passages across the pleura [12,21] and lesions strictly close to the liver may require a trajectory of the probe through liver parenchyma. For lesions developing adjacent to adrenal glands, α-blockers administration before the procedure is recommended [12,21,22]. The treatment of medial or inferior pole lesions close to the psoas muscle might be complicated by damage of genitofemoral or cutaneous lateral femoral nerves in 0.6% of cases, thus, requiring hydro-displacement or pneumo-displacement from the psoas [12,21]. If the lesion abuts the ureter, stent positioning with retrograde pyeloperfusion can reduce serious and irreversible complications, such as ureter stricture [19,20,21], and some authors state that proximity to the renal sinus could be complicated by a significant hemorrhage. If the tumor is completely endophytic, US guidance or contrast medium administration before the procedure should be considered [21]. Lastly, considering that probes should be inserted as perpendicularly as possible into the lesion to obtain the maximal technical success, the more the inclination of the probe differs from the axial plane, the more the technical difficulties are encountered [28]. As shown in Table 3, in this case series, 95 of 174 lesions (54.6%) presented greater technical complexity due to nearness to sensitive structures, with the subsequent need to use complex maneuvers such as oblique approaches, hydro-dissection, or passage of the probes through the hepatic parenchyma, while a total of 89 lesions (51.1%) required a complex needle approach. The prevalence of these technical complexities are similar to other works [29]. Emptying of cystic lesions was required in two patients. In the first case, the tumor to be treated was a small solid lesion within a cyst and the aspiration of most of the cystic content was done in order to prevent a loss of treatment efficacy and to allow an easier positioning of the cryoprobes. Despite this maneuver, the lesion was still vascularized at the end of the procedure and required a second cryoablation, after which it was completely avascular and did not recur during follow-up. In a second patient, the cyst emptying was required because a voluminous simple renal cyst of 14 cm was interposed between the kidney and the flank of the patient with no possible access route for the cryoprobes. Although the emptying led to a displacement of the bowel toward the lesion with the additional need for hydro-dissection, this technical maneuver allowed a safe and effective treatment of the renal tumor with no intra-operative complications and no recurrences.

These aspects determined an extension of the procedural time with an average use of the CT room of 84 min and a median dose of 43.7 mSv per patient. The radiation exposure values are comparable to the effective doses reported in some studies [30,31] and are slightly higher than the one reported by Borgbjerg et al. [32]. The dose depends on the acquisition protocol, which provides, in addition to the preliminary volumetric CT, a variable number of further volumetric scans, focused on the area of interest and with standard dosimetric modulation, in order to accurately verify the positioning of the probes and always be able to obtain quality oblique reconstructions. In relation to the total value of radiation dose, it weighs the absence of iterative reconstructions not available in our CT but present in new generation CTs.

As reported in literature [32], radiation exposure in the PCA procedure itself accounts for only a limited amount of the total radiation, with most radiation doses due to the follow-up regime. In our center, to avoid further exposure, we use a follow-up protocol based on MRI, reserving CT scans only for patients with severe CKD or contraindications to MRI with a massive dose saving, and a sensibility and specificity even higher than CT.

In this study, complications occurred in 52 treated lesions out of the total number (29.8%). This result is only slightly higher compared to one study reporting a complications rate between 8% and 25% of cases [10]. Nevertheless, when considering the major part of the literature, attesting a complication rate between 3% and 15% of cases [2,7,18,19,33,34], our results appear significantly higher. However, even though apparently discouraging, if the type of complications that occurred is taken into account, it is evident how the largest part (46/52) was grade I Clavien-Dindo complications, while only a minor part (6/52) is represented by grade II complications. Specifically, one patient had an allergic reaction to contrast medium and required intravenous administration of systemic antihistamines. Two patients had an important perirenal hematoma with development of anemia that required blood transfusions. Two patients had infectious complications. One developed hematuria and fever and required antibiotics. The other had a small pneumothorax after the procedure and was readmitted 6 days after discharge because of a nosocomial pneumonia that required antibiotics. One patient developed an arterio-venous fistula with formation of a pseudoaneurysm of 8 mm, which self-resolved and disappeared during follow-up. Of note, in this case, the treated tumor was very close to the renal artery and vein. Clavien-Dindo complications grade 3 to 5 have never occurred. The distribution of adverse events in this study is truly interesting since, in front of a meaningful higher rate of complications, there were no serious events requiring medical intervention while most of the authors, on the contrary, report some complications of grade 3 or even more in their series [10,15,18,21,35,36]. Some authors have chosen not to report complications below grade 3, evidently considering them not significant [7,29]. One of the most significant advantages of cryoablation is the natural anesthetic effect of ice. In fact, several studies demonstrated comparable outcomes between cryoablation performed with general anesthesia and cryoablation with local anesthesia [14]. The greater number of complications could be explained generally by the fact that most of the procedures were performed under local anesthesia or with mild conscious sedation with the possibility of slight movements by the patient, while the absence of major complications may be related to the experience of the operators. On the other hand, when the patient was awake, we were able to better monitor the absence of neurological complications from genitofemoral nerve injury, as suggested by de Kerviler et al. [14]. The proximity to the calyceal structures of the renal sinus or ureter has never been a real problem, likely due to a certain resistance of the walls of the excretory tract against the cold [21]. In this case series, a total of two patients had a stent placed for nephroprotective purposes before the treatment of lesions very close to the renal sinus. Only in one of them is there an irrigation with lukewarm saline that was performed during the procedure. There were no adverse events to the excretory tract except for a single case of mild hematuria, which resolved spontaneously. Despite the fact that, in the literature, the possibility of lesions to the excretory tract has been demonstrated in animal models [37], in vivo, they appear to be extremely rare events [7,19,38].

A technical success, referred to as the complete covering of the lesion by the ice ball on the first post-procedural scan, was achieved in 171 of 174 lesions (98.3%). These results compare favorably with the 94% reported in the study by Cronan et al. [8] and is in line with the rate of 98.9% reported in a recent series by Lim et al. [39]. In general, this outcome depends on technique-related factors like probe positioning, correct completion of the cycles, and visual control of the ice ball [10]. Nevertheless, technical success relies on some parameters that have to be evaluated prior to the procedure to guarantee the best technical execution. First of all, tumor dimension is the most important factor determining technical success and is a meaningful predictive factor of complications, such as hemorrhaging [18,21,22]. As stated before, the ideal lesion size to be thermally treated is less than 3 cm. However, cryoablation has demonstrated a good performance compared to radiofrequency ablation even in larger lesions, thanks to the possibility of using multiple probes and the direct visualization of the ice ball. In this series, the mean tumor size was 2.25 cm, ranging from 0.5 cm to 5.5 cm with nine masses larger than 4 cm. The average lesion size was lower than other series [7] and this may have affected the technical success rate.

We achieved a primary technical efficacy, intended as the success of the procedure with complete necrosis of the lesion at the follow-up imaging, in 162 of 170 lesions (95.3%) and a secondary technical efficacy of 98.2%, data in line with the literature, where a primary efficacy of 96% is reported, increasing up to 98% when retreats are included [2,7,19,40]. The details of the eight procedures with incomplete necrosis are reported below. In one procedure, there was a malfunction of a cryoprobe, which was unable to generate the ice ball. Subsequently, a mandatory operating pre-test for cryoprobes was introduced to avoid this type of inconvenience. In the second case, there was a problem of inadequate isolation of the cryoprobe that caused some low-power electrical discharges with a consequent focal pain. Thanks to local anesthesia, the patient was able to alert the operators and the procedure was interrupted. In another case, a very elderly patient treated with local anesthesia only was not able to provide adequate collaboration for the entire duration of the procedure, especially with regard to inspiratory apneas, and this led to an incomplete treatment of the lesion. The other cases of ineffective treatments were, respectively, a patient with polycystic kidneys, a patient with intra-cystic solid portions, a patient with a lesion very close to the renal sinus, and two patients with a tumor that required a complex approach but appeared to be well covered by the ball of ice at the end of the procedure.

Among treated lesions, 19% resulted to be benign at the histological examination. This result is an encouraging support to our approach in determining which lesions should be cryoablated, since, in the literature, it is stated that, up to 37% of cryoablated lesions, emerge to be benign at the histological evaluation [12]. However, even if significantly lower compared to that of the literature, this result is an additional confirmation that a non-negligible part of the lesions treated with cryoablation are actually benign, likely suggesting a limited role of imaging in distinguishing benign from malignant lesions. This is a perspective partly recognized by Tuncali et al. [41]. The limitations of imaging studies could be related to the appearance of some types of benign lesions that may be similar to RCC and, therefore, difficult to distinguish, such as cystic ones, angiomyolipomas with minimal fat, and oncocytomas. The total number of lesions not subjected to biopsy or with an inconclusive result were 36.2% of the total. One of the most important inconveniences of histological examination is the significant percentage of inconclusive biopsies, ranging from 0% to 21% of the collected specimens, which may be related to a large amount of necrosis or blood in the tissue sample [4], but also to the small dimension, the endophytic development of the lesion, or the poor collaboration of the patient. Consequently, some authors recommend the ablation of suspected lesions despite the histological result [16,17]. Of course, benign lesions will not require oncologic surveillance.

Recurrence-free survival has been estimated to be 100% globally at one year. This is in line with the majority of the studies, which identify a recurrence-free survival between 97% and 100% [2,10], and it is higher compared to other ones stating a local recurrence-free survival of 80% at one year [42]. Recurrence-free survival at three years was 95.3%, which is consistent with the average of 97%–98% at three years reported in the literature [17,35] and higher if compared to some studies showing a local recurrence-free survival of 79% at three years [42]. When comparing longer-term outcomes, the results are still in line with the literature. This study showed a recurrence-free survival of 88.6% at five years, which is contrary to what is affirmed in literature, showing an average of 94% at five years [35], but definitely coherent with other authors demonstrating a recurrence-free survival from 85% to 86% at five years [16,34,43]. The observed overall survival was 96.3%, 90.1%, and 78.4% at one, three, and five years, respectively. The current literature reports three years OS ranging from 88% to 93.2% [7,17], and five years OS ranging from 82% to 87% [7,16,34,42] with one study reporting a longer five years OS of 97.8% [19]. The significantly high CCI scores of our population, in addition to the age that was older compared to other series, determined by the preferential referral of these patients to cryoablation rather than surgery, may have contributed to a lower survival from all-cause mortality in the long-term.

This study showed a local recurrence rate of 2.9% at one year, of 2.9% at three years, and of 1.8% at five years, appearing consistent with some studies establishing a local recurrence rate between 0% and 5% in the first three years of follow-up [7,10,12,17,29] and significantly lower compared to others that report recurrence rates ranging from 7% to 16% [6,9,16,42,44,45] at three years. Similarly, long-term results are strongly encouraging, showing a local recurrence at five years of 1.8% against the 3% evident in some studies [8,46] and even 13% in other series [16,42,43].

This study has some limitations. First of all, this is a single-center-based study and the retrospective nature of it might have induced some selection bias. Furthermore, a non-negligible part of the lesions was not characterized with the histological examination. The patient cohort was small and, because the imaging follow-up strictly relies on individuals’ compliance, some patients have been lost, partially limiting the significance of longer-time results.

## 5. Conclusions

CT-guided percutaneous cryoablation of renal masses performed by expert interventional radiologists has proven to be a safe and effective technique in the treatment of suspicious lesions in our series with no major complications. The multidisciplinary discussion plays a fundamental role in choosing the best therapeutic approach for each patient and for the high number of lesions that remain undetermined because of a non-diagnostic or non-executable biopsy. There are several technical aspects to be taken into consideration to perform the procedure on lesions that require a complex approach, which can affect the time of occupation of the CT room and the delivered dose. The long-term oncological results are encouraging and in line with the major case series in the literature.

## Figures and Tables

**Table 1:** Characteristics of included patients.

Characteristics of Patients	All Patients (*n* = 138)	Patients with Biopsy Proven Renal Cell Carcinoma (*n* = 70)	Patients with no Biopsy Proven Renal Carcinoma (*n* = 68)	*p*-Value
**Age (years)**				
Mean (SD)	73 (10)	73 (10)	73 (8)	0.78
Median (Min-Max)	75 (43–89)	74 (43–89)	77 (45–87)	
**Gender**				
Male (*n*, %)	97 (70.3%)	52 (74.3%)	45 (66.2%)	
Female (*n*, %)	41 (29.7%)	18 (25.7%)	23 (33.8%)	0.35
**Single Kidney (*n*, %)**	11 (8.0%)	4 (5.7%)	7 (10.3%)	0.32

SD: Standard Deviation.

**Table 2:** Characteristics of treated lesions.

Characteristics of Lesions	All Lesions (*n* = 174)	Biopsy Proven Renal Cell Carcinoma (*n* = 78)	No Biopsy Proven Renal Carcinoma (*n* = 96)	*p*-Value
**Tumor Dimension (mm)**				
Median (Min-Max)	22.5 (5–55)	24.5 (10–55)	21.0 (5–50)	**0.005**
**Location (*n*, %)**				
Anterior	53 (30.5%)	25 (31.1%)	28 (29.2%)	
Posterior	97 (55.7%)	41 (52.6%)	56 (58.3%)	0.73
X	24 (13.8%)	12 (15.4%)	12 (12.5%)	
Lateral	111 (63.8%)	51 (65.4%)	60 (62.5%)	0.69
Medial	63 (36.2%)	27 (34.6%)	36 (37.5%)	
**Nearness to structures (*n*,%)**				
Ureter or renal pelvis	13 (7.5%)	7 (9.0%)	6 (6.3%)	0.50
Diaphragm or interposed costophrenic recess	15 (8.6%)	9 (11.5%)	6 (6.3%)	0.22
Abdominal wall/Psoas muscle	31 (17.8%)	17 (21.8%)	14 (14.6%)	0.22
Spleen	6 (3.5%)	1 (1.3%)	5 (5.2%)	0.22
Adrenal glands	3 (1.7%)	2 (2.6%)	1 (1.0%)	0.44
Renal artery, renal vein, inferior vena cava	17 (9.8%)	8 (10.3%)	9 (9.4%)	0.85
Bowel	23 (13.2%)	13 (16.7%)	10 (10.4%)	0.23
Cysts	20 (11.5%)	8 (10.3%)	12 (12.5%)	0.64
**Renal Score**				
Median (Min-Max)	7 (4–11)	7 (4–11)	7 (4–10)	0.21
**Renal Score (*n*,%)**				
Low (4–6)	68 (39.1%)	25 (32.1%)	43 (44.8%)	
Moderate (7–9)	94 (54.0%)	49 (62.8%)	45 (46.9%)	0.10
High (10–12)	12 (6.9%)	4 (5.1%)	8 (8.3%)	
**Histology (*n*, %)**				
Benign	33 (19%)			
Indeterminate	63 (36.2%)			
Biopsy not performed (n)	40 (23%)			
Histology inconclusive (n)	23 (13.2%)			
Malignant	78 (44.8%)	-	-	-
**Previous cryoablation site recurring lesion (*n*,%)**	16 (9.2%)	5 (6.4%)	11 (11.5%)	0.25

**Table 3:** Characteristics of cryoablation treatment.

Characteristics of Procedures	All Lesions (*n* = 174)	Biopsy Proven Renal Cell Carcinoma (*n* = 78)	No Biopsy Proven Renal Carcinoma (*n* = 96)	*p*-Value
**Patient position (*n*, %)**				
Prone	152 (87.4%)	66 (84.6%)	86 (89.6%)	0.25
Supine	2 (1.2%)	2 (2.6%)	0 (0.0%)	
Lateral decubitus	20 (11.4%)	10 (12.8%)	10 (10.4%)	
**Hydro-displacement from colon and bowel (*n*, %)**	13 (7.5%)	9 (11.5%)	4 (4.2%)	0.07
**Nearness to structures (*n*, %)**	95 (54.6%)	47 (60.3%)	48 (50.0%)	0.18
**Needles with a complex approach (*n*, %)**	89 (51.1%)	43 (55.1%)	46 (47.9%)	0.34
**Number of cryoprobes** **Median (Min-Max)**	2 (1–8)	2 (1–8)	2 (1–5)	0.15
**Trans-hepatic access (*n*, %)**	1 (0.6%)	1 (1.3%)	0 (0.0%)	-
**Ureteral stent (*n*, %)**	2 (1.1%)	1 (1.3%)	1 (1.0%)	0.87
**Procedural time (min)**				
Median (Min-Max)	84 (40–153)	85 (57–153)	84 (40–153)	0.55
**Radiation Exposure (mSv)**				
Median	43.7	37.5	31.3	0.68
**Technical success (*n*, %)**	171 (98.3%)	76 (97.4%)	95 (98.9%)	0.59
**Primary treatment efficacy** **(*n*/total lesions, %)**	162/170 (95.3%)	73/78 (93.6%)	89/92 (96.7%)	0.55
**Secondary treatment efficacy** **(*n*/total lesions, %)**	167/170 (98.2%)	78/78 (100.0%)	89/92 (96.7%)	0.31
**Overall complication rate (*n*, %)**	52 (29.8%)	26 (33.3%)	26 (27.1%)	0.37
Clavien-Dindo I	46 (26.4%)	22 (28.2%)	24 (25.0%)	0.76
Clavien-Dindo II	6 (3.4%)	4 (5.1%)	2 (2.1%)	0.50
**Hospitalization (days)**				
Median (Min-Max)	1 (0–7)	1 (1–7)	1 (0–5)	0.40

## Data Availability

The data presented in this study are available on request from the corresponding author. The data are not publicly available due to privacy restrictions.
